# PRISM: recovering cell-type-specific expression profiles from individual composite RNA-seq samples

**DOI:** 10.1093/bioinformatics/btab178

**Published:** 2021-03-15

**Authors:** Antti Häkkinen, Kaiyang Zhang, Amjad Alkodsi, Noora Andersson, Erdogan Pekcan Erkan, Jun Dai, Katja Kaipio, Tarja Lamminen, Naziha Mansuri, Kaisa Huhtinen, Anna Vähärautio, Olli Carpén, Johanna Hynninen, Sakari Hietanen, Rainer Lehtonen, Sampsa Hautaniemi

**Affiliations:** Research Programs Unit, Research Program in Systems Oncology, Research Programs Unit, Faculty of Medicine, University of Helsinki, FI-00014 Helsinki, Finland; Research Programs Unit, Research Program in Systems Oncology, Research Programs Unit, Faculty of Medicine, University of Helsinki, FI-00014 Helsinki, Finland; Research Programs Unit, Research Program in Systems Oncology, Research Programs Unit, Faculty of Medicine, University of Helsinki, FI-00014 Helsinki, Finland; Department of Pathology, University of Helsinki and HUSLAB, Helsinki University Hospital, FI-00014 Helsinki, Finland; Research Programs Unit, Research Program in Systems Oncology, Research Programs Unit, Faculty of Medicine, University of Helsinki, FI-00014 Helsinki, Finland; Research Programs Unit, Research Program in Systems Oncology, Research Programs Unit, Faculty of Medicine, University of Helsinki, FI-00014 Helsinki, Finland; Research Center for Cancer, Infections and Immunity, Institute of Biomedicine, University of Turku, FI-20014 Turku, Finland; Research Center for Cancer, Infections and Immunity, Institute of Biomedicine, University of Turku, FI-20014 Turku, Finland; Research Center for Cancer, Infections and Immunity, Institute of Biomedicine, University of Turku, FI-20014 Turku, Finland; Research Center for Cancer, Infections and Immunity, Institute of Biomedicine, University of Turku, FI-20014 Turku, Finland; Research Programs Unit, Research Program in Systems Oncology, Research Programs Unit, Faculty of Medicine, University of Helsinki, FI-00014 Helsinki, Finland; Research Programs Unit, Research Program in Systems Oncology, Research Programs Unit, Faculty of Medicine, University of Helsinki, FI-00014 Helsinki, Finland; Department of Pathology, University of Helsinki and HUSLAB, Helsinki University Hospital, FI-00014 Helsinki, Finland; Research Center for Cancer, Infections and Immunity, Institute of Biomedicine, University of Turku, FI-20014 Turku, Finland; Department of Obstetrics and Gynecology, University of Turku and Turku University Hospital, FI-20521 Turku, Finland; Department of Obstetrics and Gynecology, University of Turku and Turku University Hospital, FI-20521 Turku, Finland; Research Programs Unit, Research Program in Systems Oncology, Research Programs Unit, Faculty of Medicine, University of Helsinki, FI-00014 Helsinki, Finland; Research Programs Unit, Research Program in Systems Oncology, Research Programs Unit, Faculty of Medicine, University of Helsinki, FI-00014 Helsinki, Finland

## Abstract

**Motivation:**

A major challenge in analyzing cancer patient transcriptomes is that the tumors are inherently heterogeneous and evolving. We analyzed 214 bulk RNA samples of a longitudinal, prospective ovarian cancer cohort and found that the sample composition changes systematically due to chemotherapy and between the anatomical sites, preventing direct comparison of treatment-naive and treated samples.

**Results:**

To overcome this, we developed PRISM, a latent statistical framework to simultaneously extract the sample composition and cell-type-specific whole-transcriptome profiles adapted to each individual sample. Our results indicate that the PRISM-derived composition-free transcriptomic profiles and signatures derived from them predict the patient response better than the composite raw bulk data. We validated our findings in independent ovarian cancer and melanoma cohorts, and verified that PRISM accurately estimates the composition and cell-type-specific expression through whole-genome sequencing and RNA *in situ* hybridization experiments.

**Availabilityand implementation:**

https://bitbucket.org/anthakki/prism.

**Supplementary information:**

Supplementary data are available at *Bioinformatics* online.

## 1 Introduction

Precision oncology aims to identify targetable alterations based on molecular profiling of tumors (Schwartzberg et al., 2017). As cancers are heterogeneous diseases that evolve during treatment and follow-up (Aparicio and Caldas, 2013; Hanahan and Weinberg, 2011), an essential part is the use of transcriptomic data from samples collected before, during and after therapy (Karczewski and Snyder, 2018; Lin and Yang, 2019). However, a major unresolved challenge in analyzing longitudinal data is that the sample composition, i.e. the fraction of cancer, stromal and immune cells, in the patient-derived samples varies significantly, which severely hinders subsequent analyses (Aran et al., 2015).

Alleviating the sample composition issue by discarding low tumor content samples (The Cancer Genome Atlas Research Network, 2011, 2015) can bias the sampling to contain only cancer cell rich tumors and exclude samples from good-responding patients during therapy, which is detrimental in longitudinal cohorts. Current computational correction approaches are not ideally suited for precision oncology needs as they focus on either immune or stromal signatures and employ preset expression profiles (Schelker et al., 2018; Sun et al., 2019; Yoshihara et al., 2013), derive the sample composition without estimating the transcriptomic profiles (Newman et al., 2015; Wang et al., 2019), operate at a population level (Newman et al., 2019) or lack ability to adapt to patients lacking a matched single-cell data (Frishberg et al., 2019; Newman *et al.*, 2019).

To counter this, we present PRISM (Poisson RNA-profile Identification in Scaled Mixtures), which is a statistical latent variable framework for RNA-seq data. Compared with the existing methods, PRISM is unique in that it estimates both the composition and the constituent expression profiles simultaneously in individual bulk samples, a combination which was previously unmet. This is achieved by exploiting a single-cell reference, which is subject to the statistical model rather than being treated as ground truth, which allows PRISM to form adaptive profiles even for unmatched data. These estimates provide personalized expression profiles that are unbiased to changes in the sample composition, enabling tracking the tumor progression in individual patients.

We applied PRISM on 214 bulk RNA-seq samples that were longitudinally collected from homogeneously treated high-grade serous ovarian cancer (HGSOC) patients. HGSOC is the most common subtype of epithelial ovarian cancer (EOC) with only 43% five-year survival rate (Torre et al., 2018). It is one of the most genomically heterogeneous cancers, characterized by high number of structural changes (Ciriello et al., 2013), highlighting the importance of transcriptomic analysis and challenges in sample comparison. Our results show that the PRISM-estimated cell-type-specific expression profiles and cancer subtypes derived from them better predict disease progression than those of the composite raw bulk data. After validating the accuracy of the compositional estimates using whole-genome sequencing (WGS) and the cell-type specificity of expression levels using RNA *in situ* hybridization (RNA-ish) experiments, we confirmed the improved survival prediction in other cohorts and cancer types by using The Cancer Genome Atlas (TCGA) data.

## 2 Materials and methods

### 2.1 Patient and sample characteristics

The patient cohort consists of patients treated for ovarian or primary peritoneal HGSOC at Turku University Hospital between September 2010 and October 2018. All patients participating in the study gave written informed consent. The study and the use of all clinical material have been approved by The Ethics Committee of the Hospital District of Southwest Finland (ETMK) under decision number EMTK: 145/1801/2015.

**Fig. 1.**
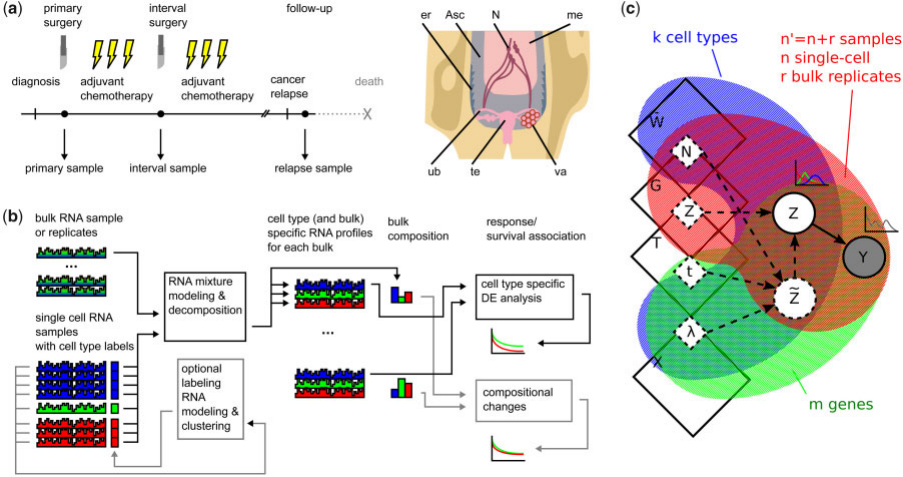
Overview of the sample collection, data analysis and the PRISM model. (**a**) Samples are collected from high-grade serous ovarian cancer (HGSOC) patients before neoadjuvant chemotherapy (120 samples), after three rounds of chemotherapy in the interval debulking surgery (60) and from relapsed cancers (20). For reference, we used single-cell RNA-seq data from eight matched samples (6312 cells). Anatomical locations of the samples are indicated as follows: Asc (ascites), LN (lymph node), Ome (omentum), Ova (ovary), Per (peritoneum), Tub (fallopian tube), Ute (uterus). (**b**) PRISM allows decomposing each bulk sample using a panel of single-cell samples, revealing the bulk compositions and expression profiles for each constituent cell type. Afterwards, differential expression or the compositional differences can be associated with patient response and survival independently. (**c**) Plate graph for the PRISM framework described by the physical constants, i.e. number of cells (*N*), sampling efficiency (*η*), expression variability ($t^{-1}$) and expression mean (*λ*) generating the latent RNA count $\tilde{Z}$ and readout *Z* for each gene and cell type in a sample. As the physical parameters are not identifiable, we parametrize the problem using mean expression (*X*), readout precision (*T*), sample scaling factor (*G*) and relative sample composition ($\bar{W}$). These parameters can be estimated from a set of mixture readouts (*Y*), which need not to be unimodal, by assuming the cell-type-specific readouts (*Z*) are scaled Poisson distributed

We acquired 214 bulk RNA sequencing samples from 61 of the patients. Of these, 120 are primary (before chemotherapy), 60 interval (after chemotherapy) and 20 relapsed tumors (after being diagnosed as recurring). The samples are from primary ovarian tumors and various sites of intra-abdominal solid metastases and ascites fluid, as detailed in the analysis. Figure 1a shows an overview of the sampling. Patient response is classified as complete response, partial response, stable disease or progressive disease according to the RECIST criteria (version 1.1) (Eisenhauer et al., 2009). The sample collection and analysis is part of the HERCULES project (http://www.project-hercules.eu/).

### 2.2 Single-cell RNA-seq sample preparation

Immediately after surgery, the HGSOC tumor specimens from our cohort were incubated overnight in a mixture of collagenase and hyaluronidase (Department of Pathology, University of Turku) to obtain single cell suspensions. Specimens were processed with a modified Fluidigm C1 protocol (Islam et al., 2014) or the standard Chromium Single Cell 3′ Reagent Kit v. 2.0 (10× Genomics) protocol for single-cell RNA sequencing with Illumina (HiSeq2000 for Fluidigm C1, HiSeq4000 or NextSeq for Chromium specimens) (Jussi Taipale Lab, Karolinska Institute or Functional Genomics Unit, University of Helsinki).

We acquired 6312 single cell profiles from 8 samples (from 7 patients and from various tissues) using the Chromium platform, and 347 cells from 8 samples (8 patients) using the Fluidigm single-cell sequencing platform. The latter were used for comparison purposes only. The single-cell samples were all matched to the bulk RNA samples but most bulk RNA samples remain unmatched. The single-cell reference need not to be matched to the bulk samples, but it needs to *span* sufficient expression state-space for PRISM to be able to adapt to unmatched profiles in the bulk samples.

### 2.3 RNA-seq preprocessing

Bulk RNA sequencing reads were preprocessed using the SePIA (Icay et al., 2016) pipeline within the Anduril framework (Cervera et al., 2019). Read pairs were trimmed using Trimmomatic (version 0.33) (Bolger et al., 2014) as follows: (i) the first 12 bases were cropped due to uneven per base sequence content; (ii) any leading bases with a quality score lower than 20 and any trailing bases with a quality score lower than 30 were removed; (iii) the reads were scanned with a 5-base wide sliding window, cutting when the average quality per base drops below 20; (iv) resulting sequences shorter than 20 bp were discarded. Trimmed reads were aligned to the GRCh38.d1.vd1 reference genome with GENCODE v25 annotation using STAR (version 2.5.2b) (Dobin et al., 2013), allowing up to 10 mismatches, and all alignments for a read were output. Gene level effective counts (we found these to be more accurate than the raw read counts) were quantified using eXpress (version 1.5.1-linux_x86_64) (Roberts and Pachter, 2013).

For the single-cell sequencing data, the raw base call (BCL) files were processed, including demultiplexing, alignment, barcode assignment and UMI quantification, with CellRanger (version 2.1.1) pipelines. The reference index was built upon the GRCh38.d1.vd1 reference genome with GENCODE v25 annotation. Single-cell transcriptomes were clustered using a shared nearest neighbor (SNN) modularity optimization based clustering algorithm implemented in Seurat (version 2.3.4) (Satija et al., 2015). PCA was selected as dimensional reduction technique in construction of SNN graph. Cell types were annotated based on acknowledged markers: epithelial cell markers: *WFDC2*, *PAX8*, *MUC16*, *EPCAM*, *KRT18*; stromal cell markers: *COL1A2*, *FGFR1*, *DCN*; immune cell markers: *CD14*, *CD79A*, *FCER1G*, *PTPRC*, *NKG7*, *CD3D*, *CD8A*.

### 2.4 Modeling RNA expression data

We assume that latent cell-type-specific RNA counts $Z_{\mathrm{ilj}}\in Z_{\geq 0}$ exist, and can be approximated by a scaled Poisson distribution, i.e. $T_{il}Z_{\mathrm{ilj}}∼P(T_{il}X_{il}{\bar{W}}_{lj}G_{j})$, where the index $i\in Z_{\left[1,m\right]}$ runs over the *m* genes, $l\in Z_{\left[1,k\right]}$ over the *k* cell types, and $j\in Z_{\left[1,n\right]}$ over the *n* samples, and $X_{il}\in R_{\geq 0}$ represents the cell-type-specific average expression profile, $T_{il}{}^{-1}\in R_{\geq 0}$ is the dispersion (specific to each cell type and gene), ${\bar{W}}_{lj}\in R_{\geq 0}$ the convex composition ($\sum_{l=1}^{k}{\bar{W}}_{lj}=1$), $G_{j}\in R_{\geq 0}$ the sample specific scale factor, and P(λ) Poisson distribution with a mean of *λ*. This approach allows capturing both biological and technical noise and accommodates either overdispersion (as commonly observed) and underdispersion (which improves stability under systematic errors) with respect to Poisson noise. The posterior of the observed $\sum_{i=l}^{k}Z_{\mathrm{ilj}}$ does not feature a closed form, but we show how to fit such models using an iterative algorithm (see Supplementary Material). Unlike previous models (McCarthy et al., 2012; Robinson et al., 2010), we are not inconvenienced by the posterior tractability and account for the discrete and heteroscedastic nature of the data (i.e. genes and cell types are not equally reliable and informative), and freely varying dispersion confers estimator robustness.

### 2.5 Decomposing bulk data using single-cell data

The model can be exploited for decomposing bulk data by considering a joint model on the bulk $y_{ir}^{\left(1\right)}\in Z_{\geq 0}$ and single-cell data $Y_{ij}^{\left(0\right)}\in Z_{\geq 0}$. Each bulk sample is analyzed separately, but could have multiple replicates, indexed by *r*, with different composition but equal expression profiles. For each bulk sample, we assume that a cell type (and bulk specific) expression profiles $\left(X_{il},T_{il}\right)$ exist, as specified in the previous section, composing the bulk and being similar to the single-cell data, i.e.:

(1) $$\begin{array}{cc} T_{il}Z_{\mathrm{ilr}}^{\left(1\right)}∼P(T_{il}X_{il}{\bar{w}}_{lr}^{\left(1\right)}G_{r}^{\left(1\right)}) & \text{st}.\;y_{ir}^{\left(1\right)}=\sum_{l=1}^{k}Z_{\mathrm{ilr}}^{\left(1\right)},\\ T_{il}Z_{\mathrm{ilj}}^{\left(0\right)}∼P(T_{il}X_{il}{\bar{W}}_{lj}^{\left(0\right)}G_{j}^{\left(0\right)}) & \text{st}.\;Y_{ij}^{\left(0\right)}=\sum_{l=1}^{k}Z_{\mathrm{ilj}}^{\left(0\right)}, \end{array}$$

where $\cdot^{\left(0\right)}$ and $\cdot^{\left(1\right)}$ refer to single-cell and bulk specific variables, respectively, $y^{\left(0\right)}$ and $Y^{\left(1\right)}$ being the single-cell and bulk data, $Z^{\left(0\right)}$ and $Z^{\left(1\right)}$ their latent random state, ${\bar{w}}^{\left(0\right)}$ and ${\bar{W}}^{\left(1\right)}$ their convex composition, and $G^{\left(0\right)}$ and $G^{\left(1\right)}$ the sample scale factors. Again, *i* runs over the genes, *l* over the cell types, *j* over the single-cell profiles and *r* over the bulk replicates (typically *r *=* *1). As *T_il_* can vary, the decomposition will weigh in the genes that are informative in discriminating the cell types. The cell-type-specific contributions ${\hat{y}}_{:lr}^{\left(1\right)}$ of the bulk $y_{:,r}^{\left(1\right)}$ can be estimated as:

(2) $$\begin{array}{c} {\hat{y}}_{\mathrm{ilr}}^{\left(1\right)}≐E[Z_{\mathrm{ilr}}^{\left(1\right)}|{\hat{X}}_{i:},{\hat{T}}_{i:},{\hat{\bar{w}}}_{:r}^{\left(1\right)},y_{ir}^{\left(1\right)}]\approx \frac{{\hat{X}}_{il}{\hat{\bar{w}}}_{lr}^{\left(1\right)}y_{ir}^{\left(1\right)}}{\sum_{l\prime =1}^{k}{\hat{X}}_{il\prime }{\hat{\bar{w}}}_{l\prime r}^{\left(1\right)}} \end{array}$$

where E[·] is expectation, $\hat{\cdot }$ are the maximum likelihood estimates of the model fit of Eq. (1), as given by Supplementary Algorithm S1, and : denotes all indices over a subscript. Further, ${\hat{\bar{w}}}_{:r}^{\left(1\right)}$ and ${\hat{G}}_{r}^{\left(1\right)}$ serve as estimators of the composition and the scale factor, respectively. This process exploits all genes and all the single-cell data, but automatically downweights the non-relevant information across the two datasets to adopt to heterogeneous settings. The relationships between the data are illustrated in Supplementary Figure S1, the relationships between the variables in Supplementary Figure S2, and a plate diagram for the full model of the decomposition process show in Supplementary Figure S3.

### 2.6 Estimating scale factors

In mixtures, the scale factors are naturally estimated as part of the deconvolution process. Meanwhile, in pure (single-component) samples, the scale factors can be estimated by considering a fraction *α* of unperturbed genes, and finding an unperturbed common subprofile $\left(x^{\left(⊤\right)},t^{\left(⊤\right)}\right)$, i.e. $t_{i}^{\left(⊤\right)}Z_{ij}∼P(t_{i}^{\left(⊤\right)}x_{i}^{\left(⊤\right)}G_{j})$ for some sparse set of genes $i\in \Omega_{j}\subseteq Z_{\left[1,m\right]}$ st. $|\Omega_{i}|=\alpha m$, revealing a global relative scaling factor *G_j_* for each single-cell sample (Supplementary Algorithm S2). Here, $\cdot^{\left(⊤\right)}$ denotes variables that are common to all samples. In the absence of any better rationale, α=50% was used.

### 2.7 Discovering constituent phenotypes

In the decomposition, the composition $W^{\left(0\right)}$ of the reference profiles (i.e. single-cell data) can be either preset or let vary freely. For more complex analyses, we also devised a hierarchical clustering process (Supplementary Algorithm S3) that exploits our model and reveals the cell types independently of the bulk. For this, $T_{il}Z_{\mathrm{ilj}}^{\left(0\right)}∼P(T_{il}X_{il}{\bar{W}}_{lj}^{\left(0\right)}G_{j}^{\left(0\right)})$ st. $Y_{ij}^{\left(0\right)}=\sum_{l=1}^{k}Z_{\mathrm{ilj}}^{\left(0\right)}$, for the single-cell data $Y^{\left(0\right)}$, but a binary composition ${\bar{W}}^{\left(0\right)}$ is built up agglomeratively. This procedure is more stable against the multiple optima than an iterative algorithm, and allows selecting the optimal number of components using statistical means, such as Bayesian information criterion (BIC) unlike general-purpose clustering algorithms (see Supplementary Fig. S4).

### 2.8 RNA *in situ* hybridization

Formalin-fixed paraffin embedded (FFPE) tissue sections were analyzed using the RNAscope Multiplex Fluorescent Reagent Kit version 2 (#323100, Advanced Cell Diagnostics). We used catalog probes (Supplementary Table S1) for the target RNAs for quantification and a positive and negative controls to verify good signal. The protocol is detailed in Supplementary Material.

For fluorescence quantification, we used CellProfiler (version 3.1.8) (Kamentsky et al., 2011) for segmentation, a Laplacian of Gaussian filter applied on a non-orthogonal basis projection for spot quantification, and cell classification based on fluorescence cosine-distance clustering (see Supplementary Material).

## 3 Results and discussion

### 3.1 PRISM: a latent statistical framework for recovering cell-type-specific expression profiles from RNA-seq data

PRISM employs a latent statistical model for composite (a mixture of multiple phenotypes) RNA-seq data, which accounts biological heterogeneity, compositional heterogeneity and sampling noise. The estimated model can be exploited for decomposing bulk RNA-seq data, finding sample specific scale factors or clustering RNA-seq data. An overview of PRISM is shown in Figure 1, details are given in Methods, and derivation in Supplementary Material. Briefly, given a bulk RNA sample, PRISM estimates the frequency and a sample specific whole-transcriptome profile for each cell type, by exploiting a labeled set of heterogeneous single-cell data for the desired cell types. The single-cell data need not be from matching tumors, but a set of sample capturing the between-patient heterogeneity in each cell type suffices. In the absence of labels, PRISM can derive a labeling through clustering (see Supplementary Fig. S5). PRISM is freely available at https://bitbucket.org/anthakki/prism/.

### 3.2 Tumor composition depends systematically on the treatment phase and the anatomical location

**Fig. 2.**
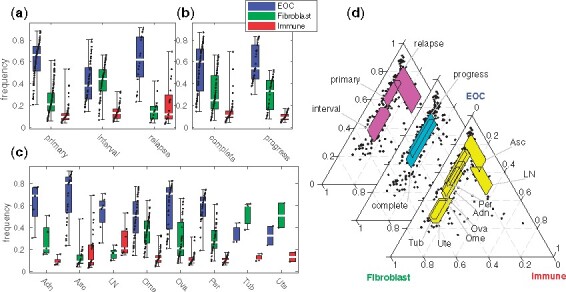
Composition of bulk RNA tissue samples in HGSOC patients. (**a**) Cancer (EOC), fibroblast and immune cell frequency by treatment phase: treatment-naive (primary), after three rounds of neoadjuvant chemotherapy (interval) or relapse. (**b**) Composition by the treatment outcome: complete response (complete) or progressive disease (progress). (**c**) Composition by the anatomical site: adnex (Adn), ascites (Asc), lymph node (LN), omentum (Ome), ovary (Ova), peritoneum (Per), fallopian tube (Tub), uterus (Ute). The boxes represent first to third quartile, white lines the medians and whiskers the data range. Black dots represent all data, jittered by their rank. (**d**) Ternary plot of the compositions. Dots represent samples and the highlighted regions box (marginal interquartile) intersections of the groups in (a), (b) and (c)

We first studied how the composition of HGSOC bulk samples varies over the treatment phase, the anatomical location and the treatment response (cf. Fig. 1a). Figure 2 shows the distribution of the PRISM-derived sample compositions. Samples taken before the treatment contain ∼70% cancer cells, while the interval samples taken after neoadjuvant chemotherapy (NACT) contain only ∼40% cancer cells, along with more fibroblasts and immune cells and the relapse samples contain more cancer and immune cells than the treatment-naive and interval samples (Fig. 2a). This is expected, as HGSOC is typically diagnosed at advanced stage with high tumor burden, and ∼80% of the patients respond well to the first-line therapy (Ledermann et al., 2013). The results reveal, however, that a direct comparison of treatment-naive and interval samples without compositional analysis is severely biased by the compositional changes.

Specifically, the fraction of cancer cells and fibroblasts vary significantly between the treatment-naive and interval samples, even when accounting for anatomical sites (*P*-value $p_{\text{rc}}<3\cdot 10^{-6}$ for no partial rank correlation in a t-test), whereas the number of immune cells does not ($p_{\text{rc}}=0.7$). Similarly, we found a significant difference between the interval and relapsed cancers ($p_{\text{rc}}<0.007$), but no difference between the primary and the relapsed samples ($p_{\text{rc}}>0.07$), when accounting for the anatomical site. We also quantified, for the first time, the impact of anatomical sites to the sample composition: omentum, ovary and peritoneum have similar composition ($p_{\text{rc}}>0.06$), when accounting for the treatment phase differences (Fig. 2c). Also fallopian tube and uterus are similar with each other, whereas the composition of the ascites samples differs significantly from the solid samples ($p_{rc}<0.0003$; Fig. 2c).

Tumor composition differences between the complete response versus progressive disease groups (Fig. 2b) are explained solely by the variations in the treatment phase (Fig. 2a) and anatomical site (Fig. 2c) of the sample ($p_{\text{rc}}>0.09$), which both contribute independent variation. Consequently, we argue that the composition of a patient bulk tissue sample is a strong confounder, but not a major predictive factor the patient response, necessitating expression profile analysis that controls for the sample composition.

### 3.3 Decomposing bulk RNA-seq data enables cell-type-specific gene expression analysis

**Fig. 3.**
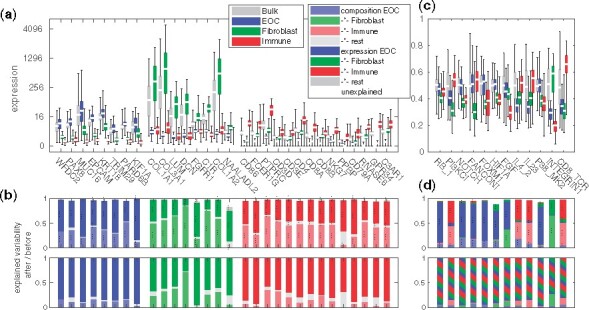
Expression in the composite bulk and the decomposed, cell-type-specific signals. (**a**) Distribution of expression levels by cell type. Box is first to third quartile, white is median, whiskers are all data. (**b**) Breakdown of the expression variability before (upper panel) or after (lower panel) the decomposition. The groups of genes represent cancer (EOC), fibroblast and immune specific genes, respectively. (**c**) The corresponding relative pathway activity using GSEA (Subramanian *et al.*, 2005) scores and (**d**) the breakdown of its variability. Two or three dots indicate significance at 0.01 and 0.001, respectively

Next, we examined the PRISM-derived cell-type-specific expression profiles in the cancer, stromal and immune cells. Figure 3a shows that the expression levels of well-known cell-type-specific genes are higher in the respective cell type (*P*-value $p_{m}<2\cdot 10^{-15}$ for equal medians in a rank-sum test), and that the cell-type-specific expression is enriched in the decomposed profiles with respect to the composite bulk ($p_{m}<0.0009$). These imply that, the composite expression signal is also diluted by the presence of non-specific signals, masking cell-type-specific phenotypic changes. The cell-type specificity of known housekeeping genes (Hsiao et al., 2001) is significantly lower than other genes with comparable expression level ($p_{m}<6\cdot 10^{-6}$), suggesting the specificity is well-founded.

We performed variance analysis (ANOVA; see Supplementary Material) of the ranked expression data to quantify the extent to which the composite expression profiles are corrupted by the sample composition. In the composite data, ∼40 to 90% ($p_{\text{rc}}<0.0003$) of the variation is explained solely by the composition, as shown Figure 3b. Interestingly, the effect varies between the genes. For instance, *KIF1A* expression has only 16% compositional effect, whereas *C1R* expression is explained by 77% by the composition, and the immune specific genes, e.g. *PPBP*, are more susceptible of having a cancer or fibroblast component. This suggests that the immune cell gene expression patterns are more dependent on the microenvironment composition than that of the other cell types. Consequently, previous analyses performed on patient tissue samples without accounting for the compositional factors likely remain useful, but may be biased toward findings in less compositionally affected gene sets.

When analyzing the PRISM-derived cell-type-specific profiles, only ∼0 to 15% of variation is explained by the composition, as shown in Figure 3b. This indicates that PRISM can eliminate the confounding effect of composition variation in the decomposed signals and enrich the sample specific signal of the constituent cell types, as intended. Further, the remaining variation is captured by the cell-type-specific decomposed expression profiles ($p_{\text{rc}}<2\times 10^{-8}$; see Fig. 3b), suggesting that the signal passing through to the decomposed cell types is both a significant explanatory factor and that it well captures the sample specificity of the original composite bulk sample.

We also verified that the cell-type specificity of expression patterns is not limited to individual genes, but is reflected in pathway activity estimates as well. We derived gene set enrichment analysis (GSEA) scores (Subramanian et al., 2005) for the NCI Pathway Interaction Database (NCI-PID) (Schaefer et al., 2009) pathways (see Supplementary Material) from both the composite and decomposed data as shown in Supplementary Figure S8. While most of the differential pathway scores appear to originate from cancer cells, a significant effect is contributed by fibroblasts or immune cells depending on the pathway. For example, the NOTCH, FOXM1 and HIF1A pathway scores appear to originate from the cancer cells (<6% from other sources); RB1, PI3KCI and FANCONI from a combination of cancer and immune cells (<4%); FGF from cancer and stroma (<2%); and IL4 and IL23 mostly from immune cells (<3%), as shown in Figure 3c and Supplementary Figure S9. Accordingly, the pathway scores using the decomposed profiles yield higher GSEA scores, indicating that the decomposition allows performing pathway analysis at a finer level of detail, by removing the compositional variation and the nuisance cell components, as suggested by Figure 3b. The results were confirmed in the TCGA ovarian cancer dataset (The Cancer Genome Atlas Research Network, 2011) (see Supplementary Material).

### 3.4 Validation of the composition estimates

To verify that the composition is accurately estimated, we compared the PRISM estimates with estimates derived from whole-genome sequencing (WGS) data. Supplementary Figure S6a shows the correlation with ASCAT (Van Loo et al., 2010) purity estimates from the corresponding WGS data. The correlation is 77% (*P*-value $p_{\text{lc}}<7\cdot 10^{-17}$ for no linear correlation in a t-test). Further, we verified that the composition can be accurately estimated in other datasets and cancer types. Thus we applied PRISM on the TCGA ovarian cancer (The Cancer Genome Atlas Research Network, 2011) bulk RNA sequencing data using our single-cell data; and to the TCGA skin cutaneous melanoma (The Cancer Genome Atlas Research Network, 2015) bulk RNA sequencing data using the single-cell data from Tirosh et al. (2016) and compared with the estimates from TCGA clinical data (immunohistochemistry) (The Cancer Genome Atlas Research Network, 2011, 2015), ABSOLUTE (Carter et al., 2012) (whole-genome sequencing) and LUMP (Aran *et al.*, 2015) (methylation 450k array) from Aran *et al.* (2015) (see Supplementary Material). Finally, we verified that comparable composition estimates are obtained by using a single-cell panel derived from a different sequencing platform and when holding out the matching patients (see Supplementary Figs S13 and S16 and Supplementary Material).

### 3.5 Validation of cell-type specificity of expression profiles

**Fig. 4.**
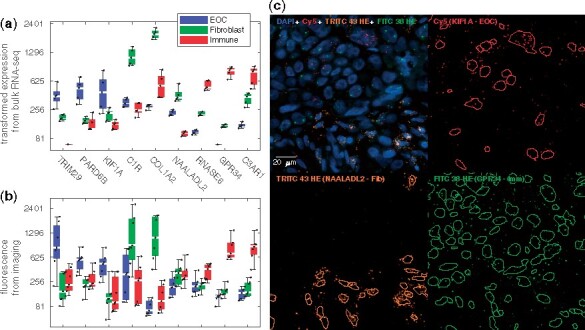
Validation of cell-type-specific expression patterns. (**a**) Predicted expression level (scaled to match the RNA-ish experiment) from the decomposed bulk RNA samples grouped by the cell type for the seven matching samples seven patients for TRIM29, PARD6B, KIF1A [cancer (EOC)], C1R, COL1A2, NAALADL2 (fibroblast), RNASE6, GPR34, C3AR1 (immune). The box denotes first to third quartile, white bar median and whiskers all data. Dots represent the samples, jittered by their rank. (**b**) The corresponding quantified fluorescence from RNA-ish measurements. (**c**) A region from the RNA-ish imaging, with split channels and our segmentation, exemplifying the cell-type specificity of the genes

We performed RNA-ish experiments to verify that the PRISM decomposed profiles are indeed expressed differentially in cancer, stromal and immune cells. For this, we used three genes for each cell type: *TRIM29*, *PARD6B*, *KIF1A* (cancer), *C1R*, *COL1A2*, *NAALADL2* (fibroblast), *RNASE6*, *GPR34* and *C3AR1* (immune). The genes were selected to have high expression in the specific cell type (Fig. 3) and a significant difference between the complete response and progressive disease groups. The validation used samples from seven HGSOC patients with matching bulk RNA-seq data, and as show in Figure 4, all the nine genes, except for *NAALADL2*, are highly expressed in the PRISM predicted cell type ($p_{m}<10^{-8}$; Fig. 4a versus Fig. 4b). The cell-type specificity in the RNA-ish experiment is also visually apparent under the microscope (Fig. 4c).

### 3.6 Decomposed RNA profiles predict patient response

The PRISM analysis revealed several genes with expression level differences between complete response and the progressive disease patients groups. The most prominent are shown in Supplementary Figure S7. Cancer specific genes *TRIM29*, *PARD6B* and *KIF1A* were found to be upregulated in the progressive disease group, while the fibroblast specific *C1R*, *COL1A2* and *NAALADL2*, and immune specific *RNASE6*, *GPR34* and *C3AR1* are downregulated in the progressive group ($p_{m}<7\cdot 10^{-8}$). In the RNA-ish data, the difference was significant for six genes (*KIF1A*, *C1R*, *COL1A2*, *RNASE6*, *GPR34* and *C3AR1*), for *TRIM29* and *PARD6B* the trend was opposite, and for *NAALADL2* was inconclusive. The trend of TRIM29 and PARD6B opposite to the general trend is due to the seven patients being a counterexample with respect to the general population: also the PRISM-derived expression the opposite pattern (83% correlation, $p_{rc}<0.003$) in these samples, while the general trend is reproduced by the TCGA ovarian cancer (The Cancer Genome Atlas Research Network, 2011) patients.

**Fig. 5.**
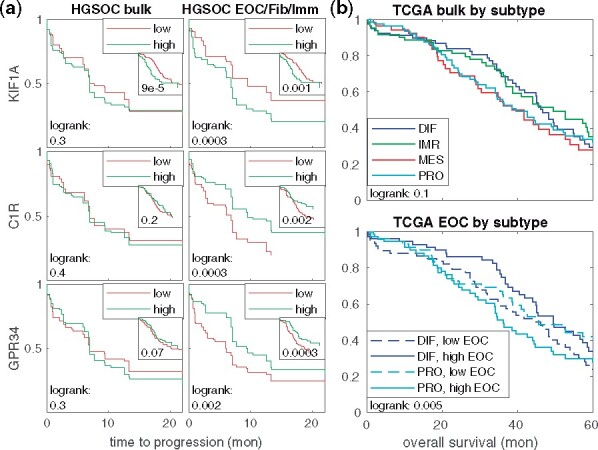
Survival association of the composite and decomposed RNA-seq data. (**a**) Time to cancer progression between groups of samples with bottom and top 50% expression of *KIF1A* [cancer (EOC)], *C1R* (fibroblast) or *GPR34* (immune) when using the composite (left) or the PRISM-derived cell-type-specific expression levels for the corresponding cell type (right). The corresponding overall survival in the TCGA ovarian cancer (The Cancer Genome Atlas Research Network, 2011) dataset are shown in the insets. (**b**) Overall survival in the TCGA ovarian cancer dataset when grouped by the subtypes derived from composite bulk data (upper panel) or the PRISM-derived cancer cell specific signal (lower panel)

For *KIF1A*, *C1R* and *GPR34*, we divided the 214 bulk RNA samples into the bottom 50% and top 50% groups by the expression level to predict the time to progression of the disease. As suggested by the differences between the complete response and progressive disease groups, we found that a high level of *KIF1A* in the cancer cell specific profile and low levels of *C1R* and *GPR34* in the fibroblast and immune specific profiles, respectively, confer less effective treatment and more rapid recurrence of the cancer. As shown in Figure 5, this difference is not visible in the composite bulk signal. We verified that a similar association exists in the decomposed TCGA ovarian cancer (The Cancer Genome Atlas Research Network, 2011) data for *KIF1A*, *C1R* and *GPR34* (*P*-value $p_{h}<0.002$ for equal hazards in a log-rank test) regarding overall patient survival (see Supplementary Fig. S15). While the trend is also visible in the composite bulk data for *KIF1A* ($p_{h}=0.0004$), the results for *C1R* ($p_{h}=0.2$) and *GPR34* ($p_{h}=0.08$) are not. In general, the survival associations are more significant for the decomposed data for the selected genes and at the whole-transcriptome scale in both ovarian cancer and in skin cutaneous melanoma (see Supplementary Figs S14 and S17 and Supplementary Material).

*KIF1A*, *C1R* and *GPR34* have not been previously associated with HGSOC survival. *KIF1A* overexpression has been associated with cancer tissue in endometrial cancer (Wong et al., 2007) and it confers docetaxel resistance in breast cancer cell lines (De et al., 2009). Peptidase S1 protein family genes, such as *C1R*, are often expressed in the stroma and endothelium of various malignant tumors (Bulla et al., 2015; Reis et al., 2018), and are associated with innate immune response activation, inducing phagocytosis, among various functions (Markiewski and Lambris, 2009; Reis *et al.*, 2018). *GPR34* is expressed primarily in specific immune cells (Schoneberg et al., 2018) and is required for adequate immune response in mice (Liebscher et al., 2011); it has been shown to be differentially expressed to the non-cancerous tissue in at least six different cancer types (Schoneberg *et al.*, 2018). The expression differences of these genes and their relevant function in other cancers warrants further study of these genes as prognostic and/or therapeutic targets.

Several studies have reported gene expression signatures in HGSOC and other cancers. As these are predominantly derived from bulk RNA-seq data, we tested their robustness in PRISM decomposed profiles. We derived HGSOC subtype estimates using the CLOVAR method (Verhaak *et al.*, 2013), which classifies the samples into differentiated (DIF), immunoreactive (IMR), mesenchymal (MES) or proliferative (PRO) subtypes from both the composite and decomposed RNA profiles. Our results indicate that within the HGSOC subtypes, the IMR subtype highly depends on the immune cell frequency alone (77% correlation; see Supplementary Fig. S10) and the MES subtype on fibroblasts (84%). DIF and PRO subtypes appear to originate from cancer cells and are more weakly correlated with the composition ($p_{\text{rc}}>0.5$), suggesting that these subtypes likely reflect phenotypic differences in the cancer cells, unlike the IMM and MES subtypes. The results were consistent between the our longitudinal and the TCGA ovarian cancer datasets (cf. Supplementary Fig. S11).

In the TCGA dataset we found that deriving the subtypes in the absence of fibroblast and immune signals yields a significantly better separation in the overall survival ($p_{h}<0.006$) than from the composite bulk data ($p_{h}=0.2$), as shown in Figure 5. To exclude the possibility that the gene expression signatures are unstable in HGSOC only, we analyzed gene expression signatures in TCGA skin cutaneous melanoma (The Cancer Genome Atlas Research Network, 2015) dataset using the expression-derived subtypes (The Cancer Genome Atlas Research Network, 2015). Here, the ‘immune’ subclass reflects mostly immune cell frequency (77% correlation, $p_{\text{rc}}<0.03$; see Supplementary Fig. S12), while the MITF-low and keratin subtypes represent likely phenotypic differences between the cancer cells. Again, after removing the confounding immune component and the compositional variation, the patient classification predicts overall survival much better ($p_{h}=0.007$ versus 0.02; see Supplementary Fig. S12).

In general, our results indicate that some of the previously reported cancer subtypes obtained by clustering composite expression data are explained by the sample composition variation alone. This is in line with a previous report in head and neck cancer (Puram et al., 2017). While the composition may be indicative of patient survival (e.g. high immune content tends to correlate with better survival), our results show that the patient response and survival can be more accurately predicted by subtyping the cell-type-specific signals separately.

## 4 Conclusion

We developed a statistical framework, PRISM, for the analysis of heterogeneous RNA mixtures, and showed how it can be exploited for extracting the composition and the bulk-adapted whole-transcriptome profiles for each constituent cell type from each individual bulk RNA sample. By analyzing 214 longitudinal HGSOC samples, we showed that the tumor composition varies systematically with the treatment phase and the anatomical location, posing a challenge in personalized transcriptomic analysis. We showed that these challenges can be overcome with PRISM, which accurately estimates cell-type-specific expression profiles, which can serve as better predictors of patient response than bulk RNA-seq data. Importantly, analysis of 308 TCGA ovarian cancer, and 474 TCGA skin cutaneous melanoma samples agreed with these findings, showing that PRISM can adapt to both different cohorts and cancer types.

The main limitation of PRISM is that a heterogeneous sample of single-cell data from each cell type involved is required for consistent performance, which can be a problem if the reference and the analysis datasets are stratified according to different criteria. However, as we have shown, good performance can be expected without matching data as long as the single-cell data is not inherently biased. This requires a single-cell reference that spans well the expression state-space of the bulk samples. Further, as a statistical method, the expression profile estimates for infrequent cell types can be inaccurate. While this permits unbiased comparison of the frequent cancer (or aggregate stromal or immune) profiles, the data might lack power for the comparison of specific infrequent subtypes of stromal and immune cells separately. These points may warrant further investigation, but we expect that the issues are mitigated in the future as single-cell cataloging efforts move forward.

Precision oncology approach calls for methods that can exploit general statistical patterns in a cohort of a heterogeneous disease, but operate reliably at the individual patient level regardless of the evolving disease state, and adapt to the specifics of that patient, to which PRISM is a response regarding whole-transcriptome analysis of bulk samples. We believe PRISM has direct applications in analyzing transcriptomic data from other diseases that stem from heterogeneous causes and sampling setting, such as other cancer types, and that analysis methods for other genomic domains can benefit from the insights of our approach.

## Supplementary Material

btab178_Supplementary_Data
